# Mapping Cellular Microenvironments: Proximity Labeling and Complexome Profiling (Seventh Symposium of the Göttingen Proteomics Forum)

**DOI:** 10.3390/cells8101192

**Published:** 2019-10-02

**Authors:** Oliver Valerius, Abdul R. Asif, Tim Beißbarth, Rainer Bohrer, Hassan Dihazi, Kirstin Feussner, Olaf Jahn, Andrzej Majcherczyk, Bernhard Schmidt, Kerstin Schmitt, Henning Urlaub, Christof Lenz

**Affiliations:** 1 Institute for Microbiology and Genetics, Georg August University, 37077 Göttingen, Germany; ovaleri@gwdg.de (O.V.); kschmit1@gwdg.de (K.S.); 2 Institute of Clinical Chemistry, University Medical Center Göttingen, 37075 Göttingen, Germany; asif@med.uni-goettingen.de (A.R.A.);; 3 Department of Medical Statistics, University Medical Center Göttingen, 37075 Göttingen, Germany; 4 Gesellschaft für Wissenschaftliche Datenverarbeitung mbH Göttingen, 37077 Göttingen, Germany; rbohrer@gwdg.de; 5 Clinic for Nephrology and Rheumatology, University Medical Center Göttingen, 37075 Göttingen, Germany; dihazi@med.uni-goettingen.de; 6 Center for Biostructural Imaging of Neurodegeneration (BIN), University Medical Center Göttingen, 37075 Göttingen, Germany; 7 Department of Plant Biochemistry, Albrecht von Haller Institute for Plant Sciences, Georg August University, 37073 Göttingen, Germany; kirstin.feussner@biologie.uni-goettingen.de; 8 Proteomics Group, Max Planck Institute of Experimental Medicine, 37075 Göttingen, Germany; jahn@em.mpg.de; 9 Büsgen Institute, Section Molecular Wood Biotechnology and Technical Mycology, Georg August University, 37077 Göttingen, Germany; amajche@gwdg.de; 10 Institute for Biochemistry, University Medical Center Göttingen, 37075 Göttingen, Germany; bernhard.schmidt@med.uni-goettingen.de; 11 Bioanalytical Mass Spectrometry, Max Planck Institute for Biophysical Chemistry, 37077 Göttingen, Germany; 12 DFG Collaborative Research Centre SFB1190 “Compartmental Gates and Contact Sites in Cells”, 37075 Göttingen, Germany

**Keywords:** proteomics, complexome profiling, proximity labeling, mass spectrometry

## Abstract

Mass spectrometry-based proteomics methods are finding increasing use in structural biology research. Beyond simple interaction networks, information about stable protein-protein complexes or spatially proximal proteins helps to elucidate the biological functions of proteins in a wider cellular context. To shed light on new developments in this field, the Göttingen Proteomics Forum organized a one-day symposium focused on complexome profiling and proximity labeling, two emerging technologies that are gaining significant attention in biomolecular research. The symposium was held in Göttingen, Germany on 23 May, 2019, as part of a series of regular symposia organized by the Göttingen Proteomics Forum.

## 1. Symposium Overview

The one-day symposium “Mapping Cellular Microenvironments: Proximity Labeling and Complexome Profiling” organized by the Göttingen Proteomics Forum (GPF) was held at the Max Planck Institute of Experimental Medicine in Göttingen, Germany on 23 May, 2019. Following the example of past GPF symposia to focus on emerging topics in proteomics research, this year’s event was co-organized by the Collaborative Research Centre SFB1190, a local research consortium that focuses on functional protein clusters located at cell compartmental gates and contact sites. Witnessed by more than 100 participants, a range of invited and local speakers presented novel or updated approaches and applications for proximity labeling and complexome profiling, techniques that complement conventional affinity-purification mass spectrometry (AP-MS) for the characterization of biologically relevant protein-protein interactions. Beyond identification of physically interacting proteins, these techniques can provide information about proteins in spatial proximity of a protein of interest (proximity labeling) or the composition, stoichiometry and apparent molecular weight of stable protein complexes (complexome profiling). Both techniques have recently gained significant attention in biomolecular research. 

## 2. Complexome Profiling

Complexome profiling was the topic of the morning session, encompassing a set of methods to characterize the composition of protein complexes on a global scale under near-native conditions. Complex proteome preparations are fractionated for the apparent molecular weight of stable protein complexes by either blue native polyacrylamide gel electrophoresis (BN-PAGE) [1] or size exclusion chromatography (SEC) using non-denaturing buffer systems [2]. Fractions are digested by endopeptidases and analyzed by label-free liquid chromatography/tandem mass spectrometry (LC/MS/MS) for their protein contents and abundance. Sets of co-migrating proteins that interact in stable complexes are then either identified by correlation analysis [3,4] or analyzed using extracted ion chromatogram correlation exploiting available information from complex compositional databases such as CORUM [2]. In addition, the rich and multidimensional data sets generated can be interrogated for migration behavior of selected sets of proteins of interest.

As first speaker, Uwe Schulte (Institute of Physiology, University of Freiburg, Germany) presented the pioneering work of his group on complexome profiling by BN-PAGE-mass spectrometry [5]. Using paraffin embedding and sub-millimeter cryo-microtome slicing, up to 368 horizontal slices per gel were cut to achieve very high apparent molecular weight resolution. To make up for the reduced thickness and, therefore, the protein content of these slices, wide-front BN-PAGE gels without discrete lanes were used. Following deparaffinization and tryptic digestion, slices were analyzed by high-resolution mass spectrometry, peptides and proteins were identified, and peak volumes for each peptide-to-sequence-match were extracted. Proteins co-migrating in stable complexes were then identified by correlation analysis. Due to the prolonged mass spectrometric analysis time required—up to 38 days of gradient time per lane—special consideration was given to normalization and data processing strategies to ensure consistent protein quantitation. Using recent datasets from rat brain mitochondrial membranes and mouse brain membrane preparations, Uwe Schulte discussed the benefits and analytical figures of merit of the approach in great detail. The high apparent molecular weight resolution, down to elution profile widths of 6.5 slices full width half maximum (FWHM), allowed for accurate correlation analysis, as well as for the detection of minor compositional subspecies of protein complexes. With high analytical effort, comprehensive information could be obtained, e.g., for oxidative phosphorylation (OXPHOS) complexes where all 90 subunits were identified, or the supercomplexes of the respiratory chain which could be visualized in high detail. Of note, the high resolution allowed for the detection and compositional analysis of so far undescribed complexes of unassigned function. Finally, in some cases, the applied normalization strategy allowed the deduction or at least verification of complex stoichiometries, as shown for the AMPA receptor core, a 0.7 MDa complex composed of subunits GluA1-4 and proteins TARP γ-8/γ-2, CNIH-2, and CKAMP44. Further examples from mouse kidney samples included complete visualization of the proteasome, the glycosyl phosphatidyl inositol (GPI) transamidase, and the Noom-Nicalin complexes. Overall, Uwe Schulte clearly demonstrated the power of BN-PAGE-based complexome profiling following thorough optimization especially of separation and data processing. 

In direct continuation, Daniel Kownatzki-Danger (Heart Research Center and Department of Cardiology & Pneumology, University Medical Center Göttingen, Germany), as a local speaker, presented an application-oriented approach where BN-PAGE-based complexome profiling was used to compare native cardiac protein complexes in membrane preparations from mouse ventricular cardiomyocytes, as well as in human ventricular tissue biopsies. BN-PAGE was previously proposed as a differential diagnostic tool to study, e.g., OXPHOS defects in cardiac disease [6]. Daniel Kownatzki-Danger and colleagues used hand-cast gels at a resolution of 60 slices to keep the required instrument time manageable. Even at this lower apparent molecular weight resolution, they were able to clearly visualize large protein complexes such as the respiratory chain subunits in mouse cardiomyocytes. Using hierarchical clustering to detect co-migrating proteins in an unbiased manner [7], they were also able to detect so far unknown interactions such as a novel RyR2/PLN/SERA2/PPP1R3A complex that was detected in the wildtype, but not in a phospholamban (PLN) knockout. Co-localization of PPP1R3A with RyR2 in mouse ventricular cardiomyocytes was confirmed by stimulated emission depletion (STED) fluorescence microscopy, highlighting the ability of the approach to detect novel and meaningful protein-protein interactions in complex data sets [8]. Moving one step further, the same experimental approach was applied to human ventricular biopsies from patients with atrial fibrillation and compared to patients with normal sinus rhythm. Complexome profiling showed clear differences in detected protein clusters, which are currently under further investigation. 

Differential comparison of multiple samples in a clinical context requires faster turnaround, restricting the degree of fractionation and the amount of instrument time available per sample. In addition, differential analysis of multiple samples demands for a very high degree of data completeness, i.e., sample-over-sample consistency in detection and quantitation of peptides. Data-independent acquisition mass spectrometry (DIA-MS) has emerged as a data acquisition strategy that addresses both of these challenges, provided that information about the protein composition of the sample exists to deal with false positive identifications [9]. As the third speaker, Isabell Bludau (Department of Biology, Institute of Molecular Systems Biology, ETH Zürich, Switzerland) presented a DIA-MS based approach termed ‘SEC-SWATH’ based on size exclusion chromatography under close-to-native conditions and Sequential Window Acquisition of All Theoretical Mass Spectra (SWATH-MS), a vendor-specific implementation of DIA-MS [2]. Instead of unbiased clustering of co-migration profiles to assign proteins to complexes, Bludau and colleagues used prior information from publicly available protein interaction databases such as CORUM [10] to infer potential protein-to-complex associations and control false positive assignments. A first comprehensive dataset generated from HEK293 lysates contained data on 4916 proteins, which could be assigned to 572 out of the 1753 protein complexes within the CORUM database at a False Discovery Rate of ≤5%. The high degree of consistency and data completeness obtained enabled the detection of sub-complexes and complex assembly intermediates, e.g., for the human proteasome. Importantly, the combination of DIA-MS with fast gradient chromatography enabled a significant reduction of MS acquisition time compared to traditional LC/MS/MS approaches. Recent data showed that a five-fold reduction of gradient length from 120 to 24 min still allowed to retain 80% of protein identifications compared to the initial study. In this manner, a complete complexome profiling experiment can be performed in a day, paving the way for medium throughput differential analysis, e.g., in clinical research contexts. Bludau used examples from HeLa cell subtypes CCL2 and Kyoto, as well as PRPF-depleted human cells to illustrate the feasibility of this approach. Finally, even proteoform-specific complex assembly was observed, highlighting the information content of the data sets. The data processing software ‘CCprofiler’ developed for the SEC-SWATH approach has been made available at https://github.com/CCprofiler/. 

## 3. Proximity Labeling

The afternoon session addressed proximity labeling techniques with biotin in living cells. In contrast to complexome analyses, only proteins localizing proximal to a bait protein (tens of nanometers apart) are covalently labeled and subsequently enriched from cell lysates. Captured proteins are then identified with either antibodies (targeted) or mass spectrometry (unbiased). Gene fusions couple bait proteins to enzymes that serve as source of reactive biotin derivatives, either to an engineered soybean ascorbate peroxidase (APEX) or to a modified biotin-ligase (BioID) in order to pursue biotin-based identification of labeled proteins. APEX requires cellular uptake of biotin-phenol and timed H_2_O_2_ activation for generation of reactive biotin-phenoxyl radicals that covalently bind aromatic amino acid residues at proximal proteins within minutes and below [11,12]. Mutated biotin ligases used for BioID experiments exude biotinyl-5’-AMP that covalently labels the ε-amino group of lysines or primary amines at N-termini of proximal proteins. Labeling times of first-generation BioID are in the range of hours. However, the procedure is non-toxic for cells and rather simple [13,14]. More active and faster biotin ligases were recently published and are currently in further development (see paragraphs below).

Oliver Valerius (Department of Molecular Microbiology and Genetics, Georg August University, Göttingen, Germany) and Lena Munzel (Department of Cellular Biochemistry, University Medical Center Göttingen, Germany) presented data from quantitative BioID approaches to characterize the head region of the 40S ribosomal subunit (hr40S) and to identify interaction partners of β-propeller-binding-polyphosphoinositides (PROPPINs) localized to endosomes, vacuoles and autophagic membranes, respectively. Both BioID projects targeted β-propeller proteins of the WD40-repeat domain family in the yeast *Saccharomyces cerevisiae*: Ribosomal Gβ-like protein Asc1/RACK1 and membrane-associated PROPPINs. PROPPIN-containing protein complexes are difficult to enrich with classical immunoprecipitation experiments according to their membranous cellular context. Studying membrane-localized bait proteins is feasible using APEX or BioID approaches, as both techniques rely on covalent protein biotin-labeling within living cells, enabling the capture of biotinylated proteins from cell lysates under denaturing conditions afterward, e.g., in the presence of membrane-solving detergents. Both BioID projects made use of Strep-Tactin (IBA GmbH)-based biotinyl enrichment, SILAC-based enrichment-quantification, and unbiased protein identification with mass spectrometry. Strep-Tactin captures enable nondestructive elution of biotinylated proteins through high biotin dosages. Accurate relative enrichment-quantification (e.g., using SILAC-MS) against the following two negative controls was emphasized as key requirement for subsequent candidate mining within lists of MS-identified proteins: 1. Biotinyl-enrichment from cells heterologously expressing the foreign biotin ligase birA* without fusion to the bait protein, to account for bait-unspecific biotinylation through birA*; 2. Biotinyl-enrichment from cells without expressing foreign birA*, to account for naturally birA*-independent biotinylated proteins. Biotin site-identification within significantly enriched proteins by mass spectrometry was considered as additional evidence for cellular bait proximity, with potential for an additional gain in structural information concerning the spatial orientation or the accessibility of the bait-ligase fusion protein.

The RACK1-proxiOME revealed ribosomal proteins known as direct neighbors from ribosome crystal structures, proving the feasibility of the experimental concept [15]. Key features of most of the other RACK1-proximal proteins were mRNA binding, mRNA turnover, and (ribosomal) protein ubiquitination and deubiquitination [16]. The explicit candidates place the Gβ-like protein spatially and functionally at the regulatory crossroads of active mRNA translation, quality control upon ribosome stalling (RQC), ribosome subunit clamping or dissociation, and as last-resort response ribophagy. In order to investigate an expected heterogeneous and dynamic nature of hr40S composition at different cellular sites or signals, ongoing experiments aim at varying RACK1 proximities at active, stalled, preserved, or disassembling ribosomes. Split-BioID approaches applying the protein fragments complementation strategy (see paragraphs below) enable the combination of RACK1 with context-specific neighbors to illuminate proximities of subpopulations of ribosomes. Also, the RACK1-dependency in hr40S composition is studied from adjacent BioID perspectives of neighboring (ribosomal) proteins using cells with and without RACK1.

Julien Béthune (Heidelberg University Biochemistry Center, Heidelberg, Germany) and colleagues have developed a methodology that combines the proximity-labeling technique BioID with the concept of protein fragments complementation assays, referred to as split-BioID [17]. The principle of the method is that two simultaneously tagged bait proteins, fused to the complementary inactive halves of a biotin ligase, give rise to a full-size active biotin ligase only upon bait-bait interaction. Only then self-biotinylation of the bait proteins (binary interaction) and biotinylation of proximal proteins within a microenvironment (conditional proxiOME) occurs. Proteins are often part of complexes that dynamically change their protein compositions, either according to assembly/disassembly lines or according to their functional assignments. Through the application of split-BioID for investigating the miRNA-mediated silencing pathway, the Béthune group could not only clearly differentiate between two distinct Ago2-containing functional complexes—namely the RISC-loading complex (RLC, containing TRBP and Dicer) and the miRISC— but also discovered GIGYF2 as exclusive miRISC component [17]. GIGYF2 was further characterized as true mRNA-binding protein and interactor of the alternative 5’ cap-binding protein 4EHP within a translation repressor complex. GIGYF2 also interacts with the CCR4-NOT complex required for mRNA deadenylation and degradation. Thereupon, GIGYF was proposed to be involved in two distinct mechanisms of mRNA repression, one that depends on direct mRNA-target binding (4EHP-independent but CCR4-NOT-dependent), and a second one depending on an indirect recruitment to mRNA-targets, e.g., through the miRISC complex [18]. 

Beyond studying the molecular mechanisms of miRNA-mediated silencing with split-BioID and many other biochemical and genetical approaches, the group of Julien Béthune further developed smaller biotin ligases with improved labeling kinetics that can be used in the split assay format. Already published engineered smaller and/or more efficient biotin ligase variants are BioID2, originated from an *Aquifex aeolicus* biotin ligase [19]; BASU, originated from a *Bacillus subtilis* biotin ligase [20]; and turboID/miniTurbo, both obtained through directed evolution of *Escherichia coli* birA-R118S [21]. MicroID, a minimal-size biotin ligase, was recently engineered in the lab of Julien Béthune and is currently in the optimization process for the application in split-ligase assays. To test for suitability and activity of split variants, the complementary ligase halves are fused to either FKBP12 (FK506-binding protein) or FRB (FKBP-rapamycin-binding) domain. Different combinations of N- and C-terminal tagging to FKBP and FRB are generally tested. FKBP and FRB do not interact in the absence of rapamycin but form a tight ternary complex in its presence. Corresponding split-halves fused to either FKBP or FRB are then co-expressed in HeLa cells, and overall protein biotinylation is then determined in the absence or presence of rapamycin. Different labeling time periods and biotin concentrations are tested and biotinylated proteins then analyzed with Western blot experiments using Streptavidin-HRP for detection. A recent version of split-microID has proven to react fast in these assays and displayed higher biotinylation activity after two hours of labeling time than the original split-BioID after 24 h.

In the scientific context of nucleocytoplasmic transport, Ralph Kehlenbach (Department of Molecular Biology, University Medical Center Göttingen, Germany) presented data that originated from a quantitative modified APEX approach utilizing an enhanced ascorbate peroxidase 2 (APEX2)-approach to map compartment-specific protein interactions of the vesicle-associated membrane protein-associated protein B (VapB) [22]. Since VapB localizes to the ER, as well as to the inner nuclear membrane (INM), a rapamycin-dependent dimerization assay was applied to identify protein interactions that occur specifically at the INM [23]. The APEX enzyme was fused to the FRB-(FKBP-rapamycin binding) domain and an NLS-sequence while the protein of interest—in this case, VapB—was fused to the FKBP12 protein. The addition of rapamycin induced interaction of FKBP12 and FRB and, thus, the dimerization of the two fusion constructs. Consequently, supplementation with biotin-phenol and H_2_O_2_ results in APEX-mediated biotinylation of the VapB environment that is specific for its localization to the INM. Biotinylated proteins were enriched using neutravidin (deglycosylated avidin to avoid lectin enrichment), identified with mass spectrometry, and relatively quantified against essential controls using stable isotope labeling with amino acids in cell culture (SILAC).

The decision for either APEX, BioID, split-BioID or one of their variants strongly depends on the cellular system or organism used, and on the process/complex/cellular site analyzed. Proximity labeling experiments require significant method establishing and optimization efforts. The following aspects should be considered during that process:

1. Expression system or strategy for expressing the bait-enzyme fusion protein, including codon-usage.
2. Testing for functionality/localization of the fusion protein.
3. Biotin/biotin-phenol uptake, dosage requirements and subcellular localization, toxicity.
4. Enrichment-quantification strategies and negative controls (MS^1^-based label-free quantitation (LFQ), DIA-MS-based label-free quantitation, in vivo stable isotope labeling with amino acids in cell culture (SILAC), or post-digestion chemical peptide labeling).
5. Capture/elution strategies (destructive versus non-destructive) and corresponding protein digestion protocols (in-gel versus in-solution vs. on-bead).
6. Stable versus short-lived protein interactions/proximities.
7. Labeling activity versus background noise (sensitivity versus specificity).
8. Overall proximity capture (unbiased screening) versus context-specific proximity capture (targeted proximity capture).

## 4. Conclusions

Taken together, the symposium provided a balanced overview of both complexome profiling and proximity labeling approaches, two emerging mass spectrometry-based technologies in structural biochemistry that complement traditional affinity purification approaches and thus allow for a much more fine-grained study of cellular organization and cellular function. 
